# Understanding teacher emotional exhaustion: exploring the role of teaching motivation, perceived autonomy, and teacher–student relationships

**DOI:** 10.3389/fpsyg.2023.1342598

**Published:** 2024-01-08

**Authors:** Xiaoyu Wang, Li Yang, Kun Chen, Yanan Zheng

**Affiliations:** ^1^College of Education Science, Harbin Normal University, Harbin, China; ^2^College of Teacher Education, Fujian Normal University, Fuzhou, China

**Keywords:** teacher–student relationships, perceived autonomy, teaching motivation, emotional exhaustion, structural equation modeling, educational well-being

## Abstract

**Introduction:**

This research investigates the complex interplay of factors influencing teacher emotional exhaustion within the educational environment. It draws upon a diverse sample of 680 teachers from 15 primary educational institutions in various districts of Shanghai, China.

**Methods:**

The study examines the interrelationships among teacher-student relationships, perceived autonomy, teaching motivation, and emotional exhaustion. It employs validated scales to measure these constructs and utilizes Structural Equation Modeling (SEM) for analysis.

**Results:**

The analyses using SEM reveal significant associations among the variables under study. The findings indicate robust correlations between positive teacher-student relationships, perceived autonomy, and higher teaching motivation. Concurrently, these factors exhibit a negative relationship with emotional exhaustion.

**Discussion:**

Mediation analysis further unveils that teaching motivation plays a significant mediating role in the associations between teacher-student relationships, perceived autonomy, and emotional exhaustion. This emphasizes the intricate indirect effects of these constructs.

**Conclusion:**

The study underscores the multifaceted nature of these factors and their collective impact on teacher well-being within educational settings. The implications derived highlight the importance for educational institutions to prioritize interventions fostering positive teacher-student relationships, autonomy-supportive environments, and intrinsic motivation among educators. These interventions aim to alleviate emotional exhaustion and enhance teacher well-being.

## Introduction

Education transcends the mere transmission of knowledge; it intricately involves the cultivation of meaningful connections within the classroom (Skaalvik and Skaalvik, 2020). Central to this educational landscape is the teacher–student relationship, a focal point in academic research renowned for its profound impact on academic outcomes and educator well-being (Roorda et al., 2011; Fabris et al., 2023). This relationship, woven with trust, support, communication, and mutual respect, embodies multifaceted interactions between educators and students (Pianta, 1999; Pianta and Hamre, 2009; Taxer et al., 2019). Its significance reverberates in fostering student engagement, motivation, and academic achievements, creating an environment where students feel valued and empowered to excel (Baker et al., 2008; Olivier et al., 2023).

Notably, the teacher-student relationship profoundly influences educator well-being and professional experiences (Tian et al., 2022). Positive relationships correlate with heightened job satisfaction, reduced stress levels, and a sense of fulfillment among educators (Pianta, 1999; Corbin et al., 2019). Conversely, strained relationships can lead to amplified teacher burnout and emotional exhaustion (Baker et al., 2008). This study explores two pivotal components: teacher autonomy and teaching motivation, which significantly influence educators’ well-being and instructional effectiveness.

Teacher autonomy encapsulates educators’ perceived independence and decision-making authority within their professional roles (Skaalvik and Skaalvik, 2014). Research has affirmed its substantial influence on job satisfaction, commitment, and overall well-being, empowering teachers and fostering a conducive teaching atmosphere (Dymoke and Harrison, 2006; Parker, 2015). Teaching motivation, the impetus driving educators’ passion and dedication, significantly impacts instructional quality and student outcomes (Kunter et al., 2011). While intrinsic motivation fosters job satisfaction and fulfillment, reduced motivation can lead to emotional exhaustion, affecting teacher-student interactions and educational outcomes negatively (Van den Berghe et al., 2013; Skaalvik and Skaalvik, 2016; Sato et al., 2022).

Amidst the complex educational dynamics, the integration of Self-Determination Theory (SDT) offers a comprehensive lens to comprehend motivational aspects and well-being among educators (Deci and Ryan, 2000). SDT underscores three psychological needs—autonomy, competence, and relatedness—that intrinsically drive individuals (Vansteenkiste et al., 2004; Ryan and Deci, 2014; Tilga et al., 2021). Autonomy represents teachers’ control over their work, fostering competence and relatedness, crucial for shaping well-being and motivation in educational settings (Deci et al., 2001; Skaalvik and Skaalvik, 2014). This study adopts SDT’s principles to investigate teacher-student relationships, perceived autonomy, teaching motivation, and their collective impact on emotional exhaustion among educators. By exploring these interconnected factors, this research seeks to illuminate pathways fostering positive relationships, autonomy, and motivation among teachers, essential for mitigating emotional exhaustion and creating conducive learning environments for students.

## Literature review

### Teacher emotional exhaustion

Teacher emotional exhaustion stands as a fundamental dimension of occupational burnout within the educational landscape, characterized by a profound depletion of emotional resources due to chronic stressors associated with the teaching profession (Chang, 2009; Maslach and Leiter, 2016; Klusmann et al., 2023). This state encompasses feelings of being emotionally drained, overextended, and lacking the energy necessary to meet the profession’s emotional demands (Maslach et al., 2001). Within the triad of burnout dimensions—emotional exhaustion, reduced personal accomplishment, and a sense of detachment (Maslach et al., 2001; Mérida-López and Extremera, 2017)—emotional exhaustion specifically embodies a sense of being emotionally depleted (Cui, 2022).

The persistent strain stemming from diverse student needs, administrative pressures, and workload demands significantly contributes to this emotional drain (Kyriacou, 2001; Hui et al., 2022). Moreover, emotional exhaustion often corresponds to a perceived decline in effectiveness and accomplishment in the professional role. Teachers experiencing emotional exhaustion may feel a reduced sense of achievement and efficacy in positively impacting students’ lives or contributing meaningfully to their education (Maslach et al., 2001; Rumschlag, 2017).

This emotional state can further lead to detachment from work, students, and colleagues. Educators experiencing emotional exhaustion might display signs of cynicism, withdrawal, or disengagement from their teaching responsibilities and the educational environment (Maslach et al., 2001; Mérida-López and Extremera, 2017).

The profound significance of teacher emotional exhaustion within educational contexts is evident, given its pervasive impact on individual well-being, professional efficacy, and the overall quality of education (Yin et al., 2019; Bing et al., 2022). Numerous studies have consistently linked high levels of emotional exhaustion to adverse outcomes among educators.

For instance, emotional exhaustion correlates with decreased job satisfaction and a decline in teachers’ commitment to their profession (Maslach et al., 1996; Skaalvik and Skaalvik, 2017). Teachers experiencing high emotional exhaustion might exhibit reduced enthusiasm and dedication to their roles, potentially affecting teaching effectiveness and student outcomes (Maslach and Leiter, 1999; Mérida-López and Extremera, 2017).

Furthermore, emotional exhaustion serves as a significant predictor of turnover intentions among teachers (Hakanen et al., 2006; Haerens et al., 2022). Educators experiencing high levels of emotional exhaustion might consider leaving the profession due to overwhelming emotional demands and reduced job satisfaction (Grayson and Alvarez, 2008). Notably, emotional exhaustion has also been associated with detrimental mental health outcomes, including symptoms of depression, anxiety, and increased stress levels (Skaalvik and Skaalvik, 2017). The chronic strain of emotional exhaustion significantly compromises educators’ psychological well-being (Klusmann et al., 2016).

Understanding the multifaceted nature of emotional exhaustion and its detrimental implications is essential in devising interventions and support mechanisms aimed at alleviating burnout and fostering educators’ well-being within educational settings (Samfira and Paloş, 2021). By addressing emotional exhaustion, educational institutions can contribute to enhancing teacher satisfaction, retention, and overall quality of education.

### Teaching motivation

Teaching motivation serves as a fundamental factor influencing educators’ engagement, commitment, and overall satisfaction within the teaching profession (Watt and Richardson, 2008; Han and Yin, 2016). It encapsulates both intrinsic and extrinsic drivers that sustain educators’ enthusiasm, passion, and dedication toward their teaching roles (Kunter et al., 2011; Vermote et al., 2020).

Intrinsic motivation within the teaching context emanates from the internal desire and gratification derived from the act of teaching itself (Pelletier and Rocchi, 2016). It involves a genuine interest in facilitating student learning, deriving fulfillment from witnessing students’ progress, and experiencing satisfaction from the teaching process (Kunter et al., 2011; Barni et al., 2019). Conversely, extrinsic motivation encompasses external factors that influence educators’ engagement, such as recognition, rewards, or performance evaluations (Djonko-Moore, 2022). While external incentives can influence teachers’ behavior and performance, intrinsic motivation is considered more sustainable and conducive to long-term engagement in the teaching profession (Deci et al., 2001; Firestone, 2014; Li et al., 2023).

Teaching motivation has emerged as a significant determinant in mitigating teacher emotional exhaustion (Skaalvik & Skaalvik, 2013; Bas, 2022). Educators with higher levels of intrinsic motivation, fueled by genuine passion for teaching and a sense of purpose, exhibit lower vulnerability to emotional exhaustion (Skaalvik & Skaalvik, 2013; Firestone, 2014). Studies indicate that intrinsically motivated teachers display greater resilience in coping with the challenges inherent in the teaching profession (Hultell and Gustavsson, 2011; Liu et al., 2023). Their intrinsic drive acts as a protective factor, aiding them in navigating stressors effectively and reducing the likelihood of emotional exhaustion (Skaalvik & Skaalvik, 2013).

Furthermore, intrinsic motivation fosters a positive classroom environment characterized by enthusiasm, creativity, and engagement, leading to lower levels of emotional exhaustion among educators (Hultell and Gustavsson, 2011). Teachers experiencing a genuine sense of fulfillment from their work are more likely to sustain their energy and commitment, thereby reducing emotional exhaustion. Recognizing the significance of teaching motivation in mitigating emotional exhaustion among educators underscores the importance of fostering intrinsic motivation within the teaching profession (Heinz, 2015). Strategies aimed at cultivating intrinsic motivation among instructors might involve offering opportunities for professional development, autonomy in pedagogical approaches, and creating supportive work environments that acknowledge and nurture educators’ intrinsic drive for teaching (Watt and Richardson, 2008; Ahmadi et al., 2023).

A myriad of research studies has delved into exploring the intricate correlation between teaching motivation and emotional exhaustion among educators. For instance, Van den Berghe et al. (2013) meticulously examined the intersection of emotional exhaustion and motivation among physical education instructors, emphasizing the nuanced relationship between these vital elements from both variable-centered and person-centered perspectives.

Similarly, Skaalvik and Skaalvik (2016) investigated the prediction of engagement, emotional exhaustion, and the inclination to leave the teaching profession by exploring teacher stress and self-efficacy, underlining the critical role of stress and self-efficacy in these domains. In another vein, Skaalvik and Skaalvik (2011) explored the intricate associations among teacher job satisfaction, motivation to exit the profession, and their interlinkage with school context, sense of belonging, and emotional exhaustion, emphasizing the multifaceted nature of these connections.

Additionally, Skaalvik and Skaalvik (2020) engaged in a longitudinal study concentrating on burnout dimensions, perceived school context, job satisfaction, and motivation in teaching, illuminating the interconnected nature of burnout dimensions with both motivation and job satisfaction. Furthermore, Fernet et al. (2012) scrutinized intraindividual shifts in teacher burnout, highlighting the influence of perceived school atmosphere and motivational aspects, emphasizing the predictive capacity of motivational components in anticipating changes in burnout over time. Finally, Sato et al. (2022) examined the nexus between teacher motivation and burnout within language educators, specifically exploring the repercussions of demotivating factors, contributing insightful findings into the intricate relationship between motivation, burnout, and the impact of demotivators.

Collectively, these studies shed light on the intricate dynamics linking teaching motivation with emotional exhaustion. They underscore the multifaceted nature of these connections, highlighting the predictive prowess of motivation in shaping emotional exhaustion among educators across diverse educational landscapes.

### Teacher autonomy

Teacher perceived autonomy encapsulates educators’ subjective perception and experience regarding the level of control, self-direction, and decision-making authority they possess within their professional roles (Deci and Ryan, 2000; Gawlik, 2007; Vangrieken et al., 2017). This construct comprises several interconnected elements, including instructional decision-making autonomy, classroom management autonomy, and professional collaboration and input autonomy (Parker, 2015; Patall et al., 2022).

Teacher autonomy, a cornerstone in educators’ professional experiences, extends beyond a singular dimension and encompasses various interconnected facets that collectively shape their perceived level of control and decision-making authority within their roles (Gawlik, 2005; Tilga et al., 2017; Vangrieken et al., 2017). The existing research emphasizes the multidimensional nature of teacher autonomy, encompassing cognitive, organizational, and procedural autonomy support (Stefanou et al., 2004; Tilga et al., 2017; Koka et al., 2021).

One dimension of teacher autonomy is cognitive autonomy support, reflecting educators’ freedom in making instructional decisions tailored to students’ needs and learning styles (Lamb, 2000; Stefanou et al., 2004). This facet encompasses the latitude teachers perceive in designing curricula, selecting pedagogical approaches, and assessing student progress, fostering a sense of control over their instructional practices. Another critical dimension is organizational autonomy support, which delineates teachers’ perceived independence in managing the classroom environment effectively (Friedman, 1999; Federici, 2013). This autonomy involves decision-making regarding discipline strategies, lesson pacing, and cultivating a conducive learning atmosphere, granting teachers the freedom to implement strategies aligned with their professional judgment (Koka et al., 2021). Additionally, procedural autonomy support pertains to teachers’ opportunities for collaboration and input in decision-making processes at broader institutional levels (Wermke and Höstfält, 2014; Tilga et al., 2017). Teachers feeling valued for their insights and contributions in school or district-level policies, instructional planning, or curricular development report heightened levels of procedural autonomy (Stefanou et al., 2004).

Instructional decision-making autonomy encompasses the freedom teachers perceive in making decisions related to curriculum design, pedagogical approaches, and assessment methods (Kleinkorres et al., 2023). It reflects educators’ beliefs about having the authority to tailor their teaching practices to suit students’ needs and their instructional style (Lamb, 2000; Kunter et al., 2008). Additionally, teachers’ perceived autonomy in managing the classroom environment, such as discipline strategies, lesson pacing, and creating a conducive learning atmosphere, is a crucial element (Friedman, 1999; Federici, 2013). This autonomy entails the independence educators feel in employing strategies they deem effective in maintaining a positive learning environment (Pearson and Moomaw, 2005). Lastly, the perception of having opportunities for collaboration and input in decision-making processes at the school or district level also contributes to perceived autonomy. Teachers who feel their opinions are valued and have opportunities to contribute to policy-making or instructional planning report higher levels of autonomy (Wermke and Höstfält, 2014).

The significance of teacher perceived autonomy transcends personal satisfaction; it profoundly impacts various facets of educator well-being and educational outcomes. Studies consistently demonstrate that higher levels of perceived autonomy among teachers are associated with lower levels of emotional exhaustion and burnout (Skaalvik and Skaalvik, 2014; Collie et al., 2020). When educators feel empowered to make decisions aligned with their professional judgment, they are less susceptible to emotional drain and burnout symptoms (Salokangas et al., 2020).

Moreover, perceived autonomy fosters a sense of competence, ownership, and job satisfaction among educators (Kunter et al., 2008; Dou et al., 2017; Liu et al., 2021). Teachers perceiving higher autonomy levels report increased motivation, engagement, and commitment to their profession. This, in turn, positively influences classroom practices and student outcomes, creating a more conducive and effective learning environment (Helgøy and Homme, 2007; Lyle and Peurach, 2022). Understanding the crucial role of teacher perceived autonomy in influencing educator well-being and educational outcomes highlights the importance of fostering autonomy-supportive environments within educational institutions. Policies and practices that promote collaborative decision-making, encourage teacher input in curriculum development, and provide opportunities for professional growth and autonomy are crucial (Collie et al., 2018).

Educational leaders and policymakers can implement strategies such as offering professional development opportunities that enhance instructional autonomy, fostering a culture of shared decision-making, and recognizing teachers’ expertise in educational policy formulation. Such initiatives can contribute to a more supportive and empowering work environment, ultimately benefiting both educators and students alike (Wang and Zhang, 2014).

Several research investigations have explored the intricate connection between teachers’ perceived autonomy and emotional exhaustion. For instance, Skaalvik and Skaalvik (2014) highlighted the substantial correlation between teacher self-assurance, perceived autonomy, teacher engagement, job contentment, and emotional exhaustion. Collie et al. (2018) delved into perceived autonomy support and adaptability among teachers, underscoring its relevance to workplace fatigue, disengagement, and dedication, aligning with the job demands-resources model.

Xia et al. (2022) investigated the intermediary function of teaching autonomy in shaping school culture and teacher satisfaction in early childhood education within China, accentuating the influential role of autonomy in determining teachers’ job contentment. In another study, Gavrilyuk et al. (2013) focused their study on university educators, revealing an association between perceived autonomy and burnout syndrome, indicating the key role of autonomy in alleviating burnout among teachers. Furthermore, Pogere et al. (2019) deeply explored the stress factors experienced by teachers and their coping mechanisms, establishing substantial links between emotional exhaustion, autonomy support, and coping strategies. Their findings highlighted the critical role of autonomy support in managing stressors and reducing emotional exhaustion among educators. Lastly, Skaalvik and Skaalvik (2017) correlated teachers’ motivation with school objectives, teacher self-assurance, job satisfaction, and emotional exhaustion, suggesting that an environment fostering autonomy and bolstering self-assurance positively affects emotional exhaustion among educators.

These studies underscore the paramount importance of teacher perceived autonomy in influencing emotional exhaustion among teachers. They emphasize the need to cultivate autonomy support and self-confidence to mitigate emotional exhaustion and enhance the well-being of teachers within educational environments.

### Teacher–student relationships

Teacher-student relationships represent the emotional connection, interactions, and rapport established between educators and their students within the classroom setting (Roorda et al., 2011; Longobardi et al., 2016; Ansari et al., 2020). Characterized by trust, respect, support, and effective communication, these relationships form the bedrock of a conducive learning environment (Baker et al., 2008; Poling et al., 2022). A pivotal element involves the provision of emotional support and warmth by teachers, fostering a sense of security and belonging among students (Roorda et al., 2011). When students perceive their teachers as caring and approachable, they are more likely to actively engage in learning activities (Karpouza and Emvalotis, 2019). Establishing mutual respect and trust between teachers and students is fundamental. Teachers who demonstrate respect for their students’ perspectives and capabilities contribute significantly to a positive classroom climate (Baker et al., 2008; Garcia-Rodriguez et al., 2023). Similarly, students who trust their teachers are more open to seeking guidance and actively participating in classroom discussions (Longobardi et al., 2023).

Teachers’ pupil control ideology significantly shapes the teacher–student relationship, serving as its fundamental underpinning and defining its portrayal as positive or negative (Barfield and Burlingame, 1974). This ideology encapsulates the level of authority teachers perceive they should employ in managing student conduct, a belief system intricately intertwined with the degree of trust vested in students (Hoy, 2001). This spectrum of belief frameworks ranges from a humanistic approach, characterized by a nurturing perspective acknowledging unmet student needs as the cause of misbehavior (Samfira and Sava, 2021), to a custodial viewpoint, where maintaining order through punitive measures prevails (Samfira and Sava, 2021). The pupil control ideology’s impact transcends mere classroom management, extending to the dynamics of the educational environment (Samfira and Sava, 2021). It profoundly influences trust, respect, and effective communication within teacher–student relationships (Roorda et al., 2011). Educators aligning with an autonomy-focused pupil control ideology tend to cultivate collaborative decision-making, mutual respect, and open communication in the classroom, fostering a positive teacher–student relationship (Baker et al., 2008; Wong, 2016). In contrast, an authoritative pupil control ideology, while structuring the environment, might engender differing teacher-student dynamics that deviate from a collaborative approach (Samfira and Sava, 2021).

Clear and effective communication plays a fundamental role in fostering teacher-student relationships (Roorda et al., 2017). Teachers who communicate openly, listen attentively, and provide constructive feedback create an environment conducive to meaningful interactions and understanding (Evans et al., 2019). Extensive research underscores the profound impact of positive teacher-student relationships on various aspects of students’ academic achievement and well-being (Hamre and Pianta, 2001; Longobardi et al., 2019; Martin and Collie, 2019; Zhang et al., 2023). Students who experience supportive relationships with their teachers exhibit higher levels of engagement, motivation, and academic success (Baker et al., 2008). Moreover, these relationships act as protective factors against negative outcomes such as student disengagement, behavioral problems, and emotional distress (Roorda et al., 2011). Students who feel connected and supported by their teachers are less likely to experience feelings of alienation or isolation in the classroom (Dong et al., 2021).

Recognizing the significance of teacher-student relationships or school climate necessitates the cultivation of strategies that foster positive connections within educational settings (Greenier et al., 2023). Professional development programs can train educators in relationship-building techniques, effective communication, and empathetic engagement (Baker et al., 2008). Encouraging a supportive school culture that values and promotes positive interactions between teachers and students is equally important (Falk et al., 2022).

Recent research conducted by various researchers collectively underscores the fundamental impact of teacher-student relationships on influencing the emotional fatigue experienced by teachers. Corbin et al. (2019) unveiled a direct correlation between favorable teacher-student bonds and diminished emotional exhaustion while forecasting teachers’ sense of personal accomplishment. Highlighting the interlinked nature of these elements in teachers’ welfare, Olivier et al. (2023) emphasized the cooperative roles played by self-efficacy and teacher-student relationships in alleviating emotional exhaustion. Taxer et al. (2019) corroborated these assertions, indicating that strong teacher-student connections lead to decreased emotional fatigue among educators, with heightened enjoyment and diminished anger acting as mediating factors.

Cui (2022) underscored the prognostic value of teacher-student relationships in gauging both teachers’ emotional exhaustion and enthusiasm, emphasizing the substantial impact these relationships exert on educators’ professional well-being. Carroll et al. (2022) expanded on this comprehension by exploring internal and external elements contributing to stress and burnout among teachers. Their findings suggest the relevance of these factors in shaping the teacher-student dynamic, subsequently influencing emotional exhaustion. Additionally, Tian et al. (2022) delved into the impact of transformational leadership on teachers’ burnout, accentuating the intermediary role of social–emotional competence and teacher-student relationships in this context.

Together, these studies collectively depict a cohesive understanding of the intricate interaction between teacher-student relationships and the emotional fatigue experienced by educators. They underscore the multifaceted nature of these relationships and their significance in molding the emotional well-being of teachers within the educational realm.

### Teacher–student relationships and teaching motivation

The intricate interplay between teacher–student relationships (TSR) and teaching motivation serves as a fundamental aspect in elucidating educators’ well-being and instructional efficacy within educational settings (Eldor and Shoshani, 2016; Skaalvik and Skaalvik, 2016; Roorda et al., 2017). Positive TSR, characterized by trust, respect, support, and effective communication, operates as a catalyst, significantly influencing and elevating teaching motivation (Taxer et al., 2019; Bardach et al., 2022).

Educators experiencing a nurturing and positive connection with their students tend to exhibit heightened intrinsic motivation toward their teaching roles (Spilt et al., 2011; Roorda et al., 2017). Such profound connections foster a profound sense of fulfillment and commitment among educators, ultimately shaping their dedication and enthusiasm within the classroom environment (Poulou, 2017; Taxer et al., 2019). Furthermore, teachers engaged in meaningful interactions and feeling valued by their students are more inclined to demonstrate elevated levels of teaching motivation (Bardach et al., 2022).

Recent research has provided illuminating insights into the multifaceted impact of teacher-student relationships on diverse dimensions of teaching and educator welfare. Cui (2022) examined how teacher-student relationships forecasted teachers’ professional contentment, emotional fatigue, and passion, highlighting their substantial influence on educators’ emotional well-being and professional fervor.

Similarly, Frommelt et al. (2021) explored the correlation between teacher enthusiasm, supportive teaching methodologies, and student drive in mathematics classrooms. Their findings underscored the essential role of teacher enthusiasm and supportive teaching approaches in nurturing student motivation, emphasizing the positive impact of a teacher’s enthusiasm on students’ engagement and interest in learning.

Eldor and Shoshani’s (2016) study investigated the examination of nurturing connections within school staff and its link to teachers’ work dedication. Their research shed light on the association between empathy in staff-teacher relationships and the extent of educators’ commitment to their work, underscoring the significance of fostering interpersonal bonds within the school context to sustain teachers’ dedication.

Furthermore, Derakhshan et al. (2023) multinational study delved into the interplay among an affectionate pedagogical approach, creativity, and work commitment among language educators. Their research illuminated how an encouraging pedagogical method correlated with heightened creativity and work commitment among teachers, emphasizing the potential impact of supportive teaching techniques on educators’ well-being.

Additionally, Bardach et al. (2022) comprehensive analysis explored the impact of teachers’ psychological attributes on teaching effectiveness, welfare, retention, and interpersonal connections. Their extensive review highlighted the significant role of teachers’ psychological traits in shaping various aspects of teaching outcomes and professional associations.

In summary, these studies collectively underscore the profound influence of student-teacher relationships, teacher enthusiasm, supportive instructional methodologies, empathetic connections, nurturing pedagogical approaches, and teachers’ psychological attributes on diverse facets of teaching motivation, educator welfare, engagement, and student drive within educational contexts.

### Teacher autonomy and teaching motivation

Teacher autonomy represents a cornerstone in nurturing educators’ teaching motivation and overall well-being within educational settings (Dymoke and Harrison, 2006). The perception of autonomy in decision-making, curriculum planning, and instructional methods significantly contributes to heightened motivation among educators (Skaalvik and Skaalvik, 2014; Parker, 2015).

Empowering educators with autonomy instills a sense of ownership, intrinsic motivation, and commitment to their profession (Dymoke and Harrison, 2006; Parker, 2015). Educators afforded autonomy tend to demonstrate increased job satisfaction and enthusiasm toward teaching, profoundly impacting their overall motivation (Skaalvik and Skaalvik, 2014; Kleinkorres et al., 2023). Additionally, autonomy-supportive environments provide avenues for educators to innovate in teaching methods, fostering a sense of competence and efficacy, ultimately fueling their teaching motivation (Parker, 2015).

Understanding the synergistic relationship between teacher-student relationships, teacher autonomy, and teaching motivation underscores the intricate interplay among these constructs. It emphasizes the need for educational institutions to foster environments that nurture positive teacher-student relationships, advocate for autonomy, and promote intrinsic motivation among educators. Addressing these interconnections becomes pivotal for enhancing teacher well-being and the overall quality of educational experiences within classrooms.

The significance of teacher autonomy in educational contexts and its correlation with teaching motivation has been extensively explored in numerous studies (Collie et al., 2020). Amini and Kruger’s (2022) systematic literature review delved into the role of Iranian EFL teachers’ autonomy in self-directed learning, indicating a positive link between autonomy and educators’ motivation for self-directed learning practices.

Similarly, Fradkin-Hayslip’s (2021) study using SDT examined elementary school teachers’ perceptions of autonomy, motivation, and job satisfaction, revealing a positive relationship between teacher autonomy and both motivation and job satisfaction. It highlighted autonomy’s role in fostering teachers’ intrinsic motivation and satisfaction with their work. Also, Pearson and Moomaw (2005) study investigated the correlation between teacher autonomy and stress, work satisfaction, empowerment, and professionalism. Their findings indicated that higher levels of teacher autonomy correlated with reduced stress, increased job satisfaction, empowerment, and professional growth, showcasing the positive impact of autonomy on various aspects of teachers’ professional lives.

Moreover, Kiemer et al. (2018) explored instructional and motivational classroom discourse in connection with teacher autonomy and competence support. Their study suggested a positive relationship between teacher autonomy and instructional discourse, illustrating that an environment supporting teacher autonomy was linked to more motivational classroom discourse.

Dikilitaş and Mumford (2019) also focused on teacher autonomy development through engaging with teacher research, illuminating the significance of autonomy in teachers’ professional growth. It highlighted how autonomy in exploring teacher research positively impacted teachers’ sense of agency, motivation, and professional identity.

Collectively, these studies emphasize the positive correlation between teacher autonomy and teaching motivation. They suggest that autonomy plays a crucial role in nurturing teachers’ intrinsic motivation, job satisfaction, professional growth, and in creating an environment conducive to their self-directed learning and professional development.

## The current research

**Figure 1:**
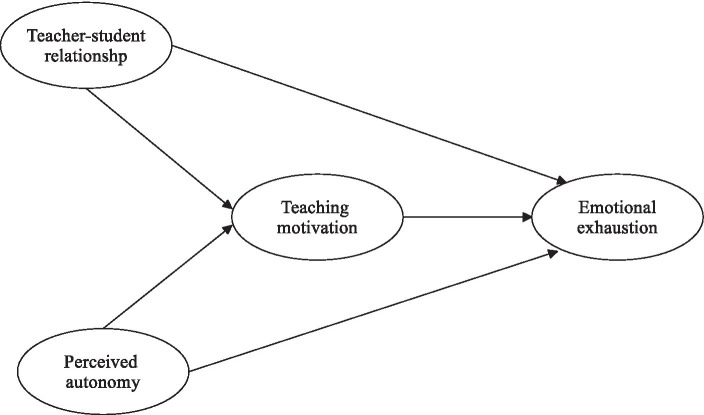
The hypothesized model.

The current study aims to investigate and analyze the intricate relationships between teacher-student relationships, perceived teacher autonomy, teaching motivation, and teacher emotional exhaustion within the educational landscape. Grounded in the extensive body of literature reviewed, this research seeks to examine the direct associations between teacher-student relationships and perceived teacher autonomy with teacher emotional exhaustion. Additionally, it intends to explore the mediating roles of teaching motivation in these relationships as depicted in the hypothesized model (see Figure 1). By delving into these dynamics, this study aims to contribute to a deeper understanding of the factors influencing teacher emotional exhaustion, thus providing valuable insights into interventions and support mechanisms within educational settings.

*H1*: Teacher–student relationship is directly related to teacher emotional exhaustion.

The existing literature provides compelling evidence supporting the direct relationship between teacher-student relationships and teacher emotional exhaustion. Studies by Corbin et al. (2019), Olivier et al. (2023), Taxer et al. (2019), Cui (2022), Carroll et al. (2022), and Tian et al. (2022) consistently demonstrate the fundamental impact of teacher-student relationships on influencing the emotional fatigue experienced by educators. Findings from these studies underscore that favorable teacher-student bonds are significantly associated with reduced emotional exhaustion among teachers. Olivier et al. (2023) highlighted the cooperative roles played by self-efficacy and teacher-student relationships in alleviating emotional exhaustion. This substantiates the proposition that stronger teacher-student connections are linked to lower levels of emotional fatigue, positively affecting teachers’ well-being.

*H2*: Perceived teacher autonomy is directly related to teacher emotional exhaustion.

The literature reviewed strongly supports the proposition that perceived teacher autonomy is directly associated with teacher emotional exhaustion. The existing literature consistently indicate that higher levels of perceived autonomy among educators are significantly correlated with reduced emotional exhaustion and burnout (e.g., Gavrilyuk et al., 2013; Skaalvik and Skaalvik, 2017; Collie et al., 2018; Pogere et al., 2019; Xia et al., 2022). Educators who feel empowered to make decisions aligned with their professional judgment are less susceptible to emotional drain and burnout symptoms (Skaalvik and Skaalvik, 2014). These findings emphasize the pivotal role of perceived autonomy in mitigating emotional exhaustion among teachers, indicating a direct relationship between autonomy and well-being.

*H3*: Teaching motivation mediates the relationship between teacher–student relationship and teacher emotional exhaustion.

The literature suggests a potential mediation effect of teaching motivation on the relationship between teacher-student relationships and teacher emotional exhaustion. Studies have highlighted the influence of teaching motivation in mitigating emotional exhaustion among educators (Skaalvik and Skaalvik, 2011, 2016, 2020; Fernet et al., 2012; Van den Berghe et al., 2013; Sato et al., 2022). Positive teacher-student relationships contribute to fostering a supportive environment, leading to higher levels of teaching motivation (Baker et al., 2008; Robinson, 2022). As evidenced, teachers who experience supportive relationships with students exhibit increased motivation and engagement. Therefore, it can be posited that teaching motivation acts as a mediator, whereby positive teacher-student relationships enhance teaching motivation, subsequently reducing emotional exhaustion among educators.

*H4*: Teaching motivation mediates the relationship between teacher perceived autonomy and teacher emotional exhaustion.

Similarly, the literature supports the hypothesis that teaching motivation mediates the relationship between teacher perceived autonomy and teacher emotional exhaustion. Studies consistently demonstrate the relationship between perceived autonomy and reduced emotional exhaustion (Gavrilyuk et al., 2013; Skaalvik and Skaalvik, 2014; Pogere et al., 2019). Higher autonomy levels contribute to increased motivation, engagement, and commitment among educators (Kunter et al., 2008; Xia et al., 2022). Given that autonomy fosters teaching motivation and influences emotional exhaustion, it is plausible to posit that teaching motivation mediates the link between perceived autonomy and emotional exhaustion among teachers.

## Methods and materials

### Participants and procedures

In this investigation, a total of 680 teachers (comprising 580 females, accounting for 85.3% of the sample) actively took part in the survey, reflecting an average age of 39.5 years (with a Standard Deviation, SD = 7.92). These educators hailed from 15 primary educational institutions situated in diverse districts within Shanghai, China. The school selection process entailed a randomized approach, targeting institutions with moderate student populations and average academic benchmarks across four administrative zones: Luwan, Xuhui, Minhang, and Jing’an. Initial outreach was directed at school principals, presenting the research objectives, culminating in consent from 15 schools to partake in the study. These schools were deemed representative of Shanghai’s typical academic landscape.

The participating instructors boasted an average tenure of 18.7 years in teaching (SD = 9.84). All educators involved in this research held full-time positions and possessed valid teaching certifications. Notably, 15.5% of them had completed postgraduate studies, while 78.9% held undergraduate degrees from accredited universities. Regarding teaching specializations, 38.2% were engaged in teaching Chinese language courses, 44.5% were dedicated to mathematics education, and the remaining instructors covered various subjects, including English, physical education, fine arts, natural sciences, and music.

The involvement of participants in this study was entirely voluntary, and prior to administering the questionnaires, written informed consent was diligently obtained from each contributor. To safeguard anonymity and ensure the confidentiality of individual identities, stringent measures were implemented, and no personal details were divulged or linked to the collated data.

### Instruments

#### Emotional exhaustion scale

The personal burnout scale (Nübling et al., 2006) was used to assess emotional exhaustion, encompassing six items that reflect components of burnout related to emotional exhaustion. Respondents rated the frequency of experiencing emotional exhaustion using a 5-point scale ranging from 1 (never) to 5 (always). For instance, a sample item asked participants, “How often do you feel emotionally drained from your work responsibilities?”

#### Teacher autonomy scale

Teacher autonomy was measured using a subset of items from the Autonomy at Work Scale, derived from the Basic Need Satisfaction at Work measure (La Guardia et al., 2000). This scale comprised five items aiming to evaluate teachers’ perceptions of autonomy within their professional setting. Participants rated their agreement with statements such as “I am free to express my ideas and opinions at work” on a 5-point Likert scale ranging from 1 (Strongly Disagree) to 5 (Strongly Agree).

#### Teaching motivation scale

Teacher motivation was assessed through a questionnaire developed by Kunter et al. (2011). This scale, initially designed to gauge the enthusiasm of mathematics instructors, consisted of 10 items. Participants expressed their level of agreement with statements such as “I genuinely enjoy teaching mathematics in this class” on a 5-point Likert scale, where 1 represented “Strongly Disagree” and 5 indicated “Strongly Agree,” allowing them to convey the extent of their motivation towards teaching.

#### Teacher–student relationship scale

The Inventory of Teacher-Student Relationships (ITSR: Murray and Zvoch, 2011) was utilized to evaluate the quality of teacher-student relationships. This inventory comprised 17 items, assessing dimensions of trust, communication, and alienation on a 4-point scale from 1 (“never”) to 4 (“always”). An example item from this scale is “I feel comfortable discussing academic difficulties with my teacher.” The mean score derived from these items indicated the overall quality of teacher-student relationships.

### Data analysis

Statistical analyses were conducted to comprehensively examine the collected data. Descriptive statistics and correlations were computed using SPSS 28.0 to offer an initial understanding of the dataset. Following this, Confirmatory Factor Analysis (CFA) was performed via AMOS 26.0 to assess the validity of constructs and model fitting. The CFA facilitated the evaluation of anticipated relationships between latent constructs and observed indicators, examining how well the proposed model aligned with the data. Subsequently, Structural Equation Modeling (SEM) was utilized to delve deeper into the hypothesized relationships among latent constructs.

Several established fit indices, as recommended by Hu and Bentler (1999), were employed to assess model fit. These indices included the χ2/df ratio, Goodness of Fit Index (GFI), Comparative Fit Index (CFI), Root-Mean-Square Error of Approximation (RMSEA), and Standardized Root-Mean-Square Residual (SRMR). A χ2/df ratio below 3, accompanied by a value of p above 0.05, signified a favorable fit. Furthermore, GFI and CFI values reaching or surpassing 0.90 indicated a favorable fit, while RMSEA values below 0.08 and SRMR values below 0.10 were deemed acceptable (Marsh et al., 2004). To ensure the strength of indirect effects, bootstrapping analyses were conducted employing 5,000 resamples, following the methodology outlined by Hayes (2009).

## Results

Initial analyses were performed to ensure the data’s suitability for subsequent modeling using SPSS 28. An initial data screening process was implemented following guidelines by Tabachnick et al. (2013) to address missing data, assess normality, and identify outliers. Given the small sample size in this study, techniques such as list-wise deletion and pair-wise deletion for handling missing data were deemed unsuitable, especially with a high amount of missing data. Consequently, the Expectation–Maximization (EM) algorithm, as recommended by Kline (2023), was employed for missing data imputation. The EM technique substitutes missing values within the dataset.

Assessment of item normality was conducted using skewness and kurtosis indices. Items displaying values beyond ±2.0 were regarded as non-normally distributed and subsequently removed from the analysis. Moreover, both univariate and multivariate outliers were identified using Z-Standardized scores and Mahalanobis *D*^2^, respectively, in accordance with Tabachnick et al. (2013). Any detected outliers were excluded from the dataset to ensure data integrity.

Then descriptive statistics were calculated for the variables of interest, including teacher-student relationship, teacher autonomy, teaching motivation, and emotional exhaustion. The means, standard deviations, and correlations among the constructs are presented in Table 1.

**Table 1:** Descriptive statistics and correlations among the constructs.

Variables	Mean	SD	1	2	3	4
1. Teacher–student relationship	3.21	0.66	1			
2. Teacher autonomy	4.11	0.78	0.32**	1		
3. Teaching motivation	3.48	0.81	0.46***	0.48***	1	
4. Emotional exhaustion	3.16	0.93	−0.44***	−0.35**	−0.61***	1

**p < 0.01, ***p < 0.001.

Table 1 displays the descriptive statistics for the variables. The mean score for teacher-student relationship was 3.21 (SD = 0.66), indicating a moderate level of perceived relationship quality between teachers and students. Teacher autonomy exhibited a mean of 4.11 (SD = 0.78), indicating a relatively high perceived level of autonomy among teachers. Teaching motivation had a mean of 3.48 (SD = 0.81), reflecting a moderate level of motivation among educators. Emotional exhaustion yielded a mean score of 3.16 (SD = 0.93), indicating a moderate level of emotional exhaustion experienced by teachers.

Correlation analysis revealed significant associations among the variables. Teacher-student relationship demonstrated a positive correlation with teacher autonomy (*r* = 0.32, *p* < 0.01) and teaching motivation (*r* = 0.46, *p* < 0.001), suggesting that a better teacher-student relationship was linked to higher perceived autonomy and stronger teaching motivation. Additionally, teacher autonomy positively correlated with teaching motivation (*r* = 0.48, *p* < 0.001), indicating a relationship where higher perceptions of autonomy were associated with increased teaching motivation. Furthermore, emotional exhaustion exhibited negative correlations with teacher-student relationship (*r* = −0.44, *p* < 0.001), teacher autonomy (*r* = −0.35, *p* < 0.01), and teaching motivation (*r* = −0.61, *p* < 0.001). These findings suggest that higher emotional exhaustion among teachers was associated with poorer teacher–student relationships, lower perceived autonomy, and decreased teaching motivation.

The subsequent phase involved the examination of the measurement model via CFA. The outcomes from the CFA displayed favorable indices indicating a good fit: χ^2^(256) = 412, CFI = 0.970, TLI = 0.955, RMSEA = 0.035 (CI 95%: 0.030–0.040), SRMR = 0.038.

The findings presented in Table 2 illustrate that all coefficients of the measures exceeded 0.70, affirming their satisfactory reliability. Additionally, composite reliabilities ranged from 0.86 (emotional exhaustion) to 0.89 (teacher autonomy). Notably, all measures exhibited significant factor loadings (*p* < 0.001) well within acceptable limits. The substantial construct reliabilities and significant factor loadings established in the results confirm the convergent validity of the model (Anderson and Gerbing, 1988).

**Table 2:** The results of measurement model.

	Items	Factor loadings	AVE	MSW	ASW	CR
Emotional exhaustion	6		0.62	0.56	0.51	0.86
Emotional exhaustion_item1		0.67				
Emotional exhaustion_item2		0.53				
Emotional exhaustion_item3		0.71				
Emotional exhaustion_item4		0.66				
Emotional exhaustion_item5		0.53				
Emotional exhaustion_item6		0.62				
Teaching motivation	10		0.72	0.61	0.55	0.88
Teaching motivation_item1		0.79				
Teaching motivation_item2		0.83				
Teaching motivation_item3		0.62				
Teaching motivation_item4		0.58				
Teaching motivation_item5		0.75				
Teaching motivation_item6		0.63				
Teaching motivation_item7		0.68				
Teaching motivation_item8		0.72				
Teaching motivation_item9		0.59				
Teaching motivation_item10		0.55				
Teacher autonomy	5		0.76	0.63	0.57	0.89
Teacher autonomy_item1		0.62				
Teacher autonomy_item2		0.55				
Teacher autonomy_item3		0.63				
Teacher autonomy_item4		0.77				
Teacher autonomy_item5		0.71				
Teacher–student relation	17		0.69	0.57	0.53	0.87
Teacher–student_item1		0.60				
Teacher–student_item2		0.83				
Teacher–student_item3		0.76				
Teacher–student_item4		0.63				
Teacher–student_item5		0.58				
Teacher–student_item6		0.75				
Teacher–student_item7		0.69				
Teacher–student_item8		0.51				
Teacher–student_item9		0.71				
Teacher–student_item10		0.66				
Teacher–student_item11		0.70				
Teacher–student_item12		0.63				
Teacher–student_item13		0.51				
Teacher–student_item14		0.56				
Teacher–student_item15		0.69				
Teacher–student_item16		0.81				
Teacher–student_item17		0.72				

AVE denotes average variance extracted; MSV stands for maximum common variance; ASV signifies average shared variance; CR denotes construct or composite reliability.

Furthermore, the average variance extracted (AVE) values surpassed 0.5, while composite reliabilities exceeded the AVE values. These outcomes reinforce the model’s convergent validity (Hair et al., 2010).

The study also undertook an evaluation of discriminant validity. Following Fornell and Larcker’s (1981) suggestion, the comparison between the Average Variance Extracted (AVE) and the squared correlation coefficients among constructs was conducted. Table 3 confirmed the presence of discriminant validity. Moreover, the examination of the Maximum Shared Variance (MSV) and Average Shared Variance (ASV) values, in combination with the AVE values, demonstrated the maintenance of discriminant validity (Hair et al., 2010). Assessment to identify common method bias was carried out using Harman’s one-factor test (Podsakoff and Organ, 1986). Results showed that the first factor accounted for 41.36% variance, below the 50% threshold, indicating that common method bias was not a significant concern in this study.

**Table 3:** Discriminant validity.

Variables	AVE	CR	1	2	3	4
1. Teacher-student relationship	0.69	0.87	0.83			
2. Teacher autonomy	0.76	0.89	0.10**	0.87		
3. Teaching motivation	0.72	0.88	0.21***	0.23	0.84	
4. Emotional exhaustion	0.62	0.86	0.19***	0.12	0.37	0.78

The values within the diagonal cells represent AVE. Meanwhile, the figures within the off-diagonal cells correspond to the squared correlation coefficients between one factor and another factor.

Furthermore, a Variance Inflation Factors (VIF) test was executed. As outlined by Neter et al. (1996), VIF determines the inflation of variances in estimated regression coefficients due to linear relationships among independent variables. In this study, VIF values ranged from 0.6443 to 0.758. Typically, a maximum VIF value exceeding 10 might indicate excessive multicollinearity influencing least square estimates (Paré and Tremblay, 2007). Hence, our findings suggest a favorable relationship among the independent constructs.

**Figure 2:**
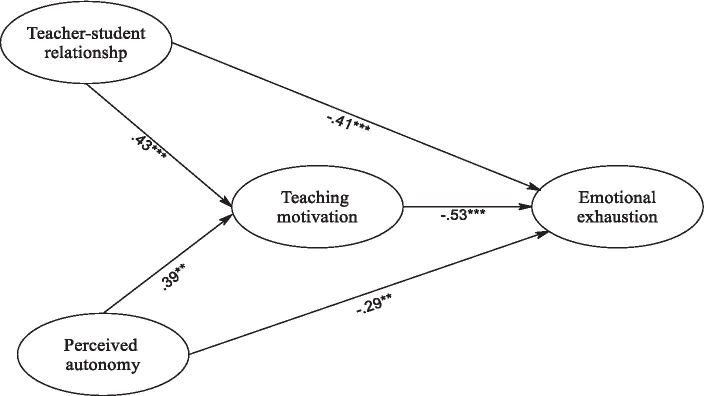
The final model of emotional exhaustion. **p < 0.01, ***p < 0.001.

Structural Equation Modeling (SEM) was utilized to investigate the proposed connections among perceived teacher affective support, perceived school climate, sense of belonging, and student engagement. Evaluation of model fit indices revealed a favorable alignment between the hypothesized model and the observed data, as evidenced by χ^2^(311) = 571, CFI = 0.975, TLI = 0.960, RMSEA = 0.033 (CI 95%: 0.029–0.038), SRMR = 0.037. A graphical representation of the proposed relationships among the latent constructs is depicted in Figure 2, illustrating all path coefficients as statistically significant. This substantiates the anticipated associations between the variables.

Furthermore, to gauge the significance of indirect effects, bootstrapping analyses employing 5,000 resamples were conducted as outlined by Hayes (2009). A summary of the bootstrapping results, encapsulating the direct, indirect, and total effects in the mediation analysis, is presented in Table 4.

**Table 4:** The SEM results.

Path	*β*	Bootstrapped 95% CI	*P*-values
*Direct effects*			
T-S relationship → exhaustion	−0.41	[−0.50, −0.32]	<0.001
Motivation → exhaustion	−0.53	[−0.62, −0.44]	<0.001
Autonomy → exhaustion	−0.29	[−0.38, −0.20]	<0.01
*Indirect effects*			
T-S relationship → motivation → exhaustion	−0.22	[−0.31, −0.13]	<0.01
Autonomy → motivation → exhaustion	−0.20	[−0.29, −0.11]	<0.01
*Total effects*			
T-S relationship → exhaustion	−0.63	[−0.72, −0.54]	<0.001
Autonomy → exhaustion	−0.49	[−0.58, −0.40]	<0.001

T-S relationship, teacher–student relationship; exhaustion, emotional exhaustion; Motivation, teaching motivation; Autonomy, teacher autonomy.

## Discussion

The current study aimed to investigate the intricate dynamics between teacher-student relationships, perceived teacher autonomy, teaching motivation, and their collective influence on teacher emotional exhaustion. The findings from this research not only corroborate but also extend the existing literature on these critical dimensions within the educational landscape.

Consistent with prior research (Corbin et al., 2019; Taxer et al., 2019; Frommelt et al., 2021; Carroll et al., 2022; Cui, 2022; Tian et al., 2022), this study reveals a significant direct relationship between teacher-student relationships and teacher emotional exhaustion. The findings emphasize that when teachers foster strong, supportive, and respectful connections with their students, they are less susceptible to emotional exhaustion (Olivier et al., 2023). This aligns with the notion that constructive teacher–student relationships act as a protective factor, mitigating the emotional drain experienced by educators (Corbin et al., 2019; Taxer et al., 2019). This finding also resonates with Roorda et al.’s (2011) assertion that positive teacher–student relationships create a conducive learning environment and are associated with lower levels of teacher stress. Teachers investing in building positive relationships often report higher job satisfaction and lower emotional exhaustion (Baker et al., 2008; Eldor and Shoshani, 2016; Lavy and Bocker, 2018). Moreover, emotional exhaustion among teachers can be linked to the emotional labor they perform daily (Maslach et al., 2001). When teachers engage in emotionally demanding interactions without the support or positive rapport with students, it can contribute to emotional exhaustion (Cui, 2022).

Additionally, findings from Carroll et al. (2022) and Tian et al. (2022) corroborate the significance of teacher-student relationships in influencing educators’ emotional well-being. Carroll et al. highlighted that sustained positive connections between teachers and students serve as a buffer against emotional exhaustion, echoing the protective nature of these relationships. Moreover, Corbin et al.’s (2019) research indicated that not only do nurturing relationships with students alleviate emotional exhaustion, but they also contribute to a more conducive classroom atmosphere, fostering a sense of psychological safety. This aligns with Olivier et al.’s (2023) argument that emotionally supportive classroom environments, facilitated by strong teacher-student relationships, alleviate emotional exhaustion. Thus, the accumulation of evidence suggests that cultivating positive teacher-student relationships not only benefits students’ academic growth but also acts as a crucial mechanism in safeguarding teachers’ emotional well-being amidst the challenges of their profession.

Moreover, in line with established research (Gavrilyuk et al., 2013; Skaalvik and Skaalvik, 2014; Collie et al., 2018; Pogere et al., 2019; Xia et al., 2022), the findings of this study reaffirm a direct relationship between perceived teacher autonomy and teacher emotional exhaustion. Teachers who perceive higher levels of autonomy in decision-making, classroom management, and professional collaboration experience lower emotional exhaustion (Skaalvik and Skaalvik, 2017; Collie et al., 2018). This resonates with the notion that autonomy serves as a buffer against the emotional strain associated with the teaching profession. When educators feel empowered to make instructional decisions aligned with their professional judgment, they are less susceptible to emotional fatigue (Skaalvik and Skaalvik, 2014; Xia et al., 2022).

Deci and Ryan’s (2000) Self-Determination Theory postulates that autonomy is a fundamental psychological need influencing intrinsic motivation and well-being. This finding is consistent with prior research emphasizing the positive impact of autonomy on reducing burnout and enhancing job satisfaction (Pearson and Moomaw, 2005; Gavrilyuk et al., 2013). When teachers perceive greater autonomy in decision-making, classroom management, and curriculum development, they experience increased feelings of competence and control, resulting in lower levels of emotional exhaustion (Collie et al., 2018; Pogere et al., 2019).

Furthermore, studies by Gavrilyuk et al. (2013) and Collie et al. (2018) highlight that the beneficial impact of perceived teacher autonomy extends beyond emotional exhaustion. Their research underscores how autonomy positively correlates with heightened job satisfaction and reduced burnout among educators. Additionally, the work of Pogere et al. (2019) accentuates that autonomy in decision-making not only reduces emotional exhaustion but also contributes to fostering a sense of empowerment and control among teachers. This aligns with Xia et al.’s (2022) emphasis on autonomy as a protective factor against emotional fatigue. Moreover, Skaalvik and Skaalvik (2014, 2017) posit that teachers who perceive higher autonomy experience lower levels of emotional exhaustion, emphasizing the pivotal role of autonomy in preserving educators’ emotional well-being. These cumulative findings reinforce the critical role of autonomy in not only mitigating emotional exhaustion but also in fostering a more satisfying and empowering professional experience for educators.

Furthermore, the present study extends the existing literature by elucidating the mediating role of teaching motivation in the relationships among the constructs. Teaching motivation emerges as a crucial factor that bridges the associations between these dimensions. Specifically, the findings suggest that teaching motivation acts as a mediator between teacher–student relationships and emotional exhaustion, as well as between perceived teacher autonomy and emotional exhaustion.

The literature has consistently highlighted the significance of intrinsic motivation in mitigating emotional exhaustion among educators (Skaalvik and Skaalvik, 2016; Han et al., 2021; Sato et al., 2022). Teachers driven by intrinsic motives, such as genuine passion for teaching and a sense of purpose, exhibit lower vulnerability to emotional exhaustion (Fernet et al., 2012; Skaalvik and Skaalvik, 2020). This study’s findings support the notion that teaching motivation, particularly intrinsic motivation, plays a pivotal role in shaping the emotional well-being of teachers, serving as a protective mechanism against emotional exhaustion within the teaching profession (Skaalvik and Skaalvik, 2011; Van den Berghe et al., 2013; Dikilitaş and Mumford, 2019). Positive teacher-student relationships characterized by trust, support, and rapport have been shown to enhance teacher motivation (Wentzel, 2009; Robinson, 2022). When teachers experience positive interactions with students, it fuels their intrinsic motivation, leading to higher job satisfaction and lower emotional exhaustion (Spilt et al., 2011; Skaalvik and Skaalvik, 2014). Positive relationships act as a source of motivation for teachers, encouraging them to invest in fostering these connections, which, in turn, reduces emotional exhaustion (Pearson and Moomaw, 2005; Baker et al., 2008).

The literature extensively underscores the significance of positive teacher-student relationships in shaping educators’ emotional well-being (Wentzel, 2009; Roorda et al., 2011). The current finding extends this understanding by elucidating that the influence of teacher-student relationships on emotional exhaustion is, in part, channeled through the motivational factors that drive educators in their profession. This finding suggests that the quality of teacher-student relationships might not only directly impact emotional exhaustion but also indirectly influence it through its effects on teaching motivation (Taxer et al., 2019). When teachers establish strong, supportive relationships with their students characterized by trust, respect, and effective communication, it positively influences their motivation towards teaching (Spilt et al., 2011). In turn, heightened teaching motivation acts as a protective factor, buffering against emotional exhaustion (Olivier et al., 2023).

Finally, it was found that teaching motivation acted as a mediator between teacher autonomy and teacher emotional exhaustion. This suggests that the influence of teacher autonomy on emotional exhaustion is, at least partially, mediated by teaching motivation. Teacher autonomy, encapsulating educators’ perceived control and decision-making authority within their professional roles (Deci and Ryan, 2000; Kunter et al., 2008), has been established as a crucial determinant of educator well-being. Teachers who perceive higher levels of autonomy tend to experience a sense of competence, ownership, and job satisfaction (Skaalvik and Skaalvik, 2021). This autonomy is intertwined with teachers’ intrinsic motivation, which stems from their internal drive and gratification derived from the act of teaching itself (Deci et al., 2001; Firestone, 2014). Consequently, heightened autonomy fosters greater teaching motivation, which acts as a protective factor against emotional exhaustion among educators (Gavrilyuk et al., 2013; Collie et al., 2018; Pogere et al., 2019).

The mediation role of teaching motivation elucidates a pathway through which autonomy’s influence extends to emotional exhaustion among teachers. This finding aligns with SDT (Deci and Ryan, 2000), emphasizing the importance of autonomy as a fundamental psychological need that directly impacts intrinsic motivation and, subsequently, well-being. The dynamic relationship between these constructs implies that fostering autonomy in educators’ professional roles may not only directly enhance their satisfaction and ownership but also indirectly buffer the detrimental effects of emotional exhaustion through the cultivation of intrinsic motivation. Educators empowered with autonomy are more likely to be intrinsically motivated, fostering a sense of purpose and engagement in their teaching practices (Kiemer et al., 2018), consequently mitigating emotional exhaustion. This intricate interplay highlights the need for educational institutions to design policies and frameworks that prioritize autonomy-supportive environments, recognizing the pivotal role autonomy plays in nurturing teachers’ intrinsic motivation and well-being.

## Conclusion

In conclusion, this study illuminates the intricate nexus between teacher-student relationships, perceived teacher autonomy, teaching motivation, and teacher emotional exhaustion within educational settings. The findings underscore the direct associations between these elements and reveal the mediating role of teaching motivation in shaping the relationship between teacher-student interactions and emotional exhaustion. The direct relationships uncovered in this study between teacher-student relationships and emotional exhaustion, as well as perceived autonomy and emotional exhaustion, highlight the critical importance of fostering positive relationships and providing autonomy-supportive environments within educational contexts. Moreover, the mediation effect of teaching motivation emphasizes its significance as a potential mechanism to alleviate emotional exhaustion among educators.

The implications drawn from this research extend far into practical avenues within the educational landscape. The revelation regarding the substantial influence of teacher-student relationships on emotional exhaustion necessitates a concerted effort to implement strategies fostering these connections. Targeted interventions through ongoing professional development programs can prioritize the cultivation of interpersonal skills, empathetic training, and refined communication strategies among educators. By enhancing these abilities, teachers can effectively nurture robust, positive relationships with their students. Additionally, instituting mentoring programs or fostering peer support networks may serve as valuable platforms for sharing best practices and strategies that promote constructive teacher-student interactions.

The explicit association between perceived autonomy and emotional exhaustion underscores the pivotal role of leadership in shaping supportive environments for educators. School administrators and policymakers bear the responsibility of cultivating autonomy-supportive cultures within educational institutions. Initiatives aimed at granting decision-making autonomy in teaching methodologies, curriculum design, and assessment approaches are vital. These efforts, complemented by ample resources, mentorship opportunities, and administrative support, can significantly mitigate the risk of burnout among teachers. Strengthening communication channels and involving educators in decision-making processes further fortifies autonomy-supportive environments, nurturing a sense of empowerment and reducing emotional exhaustion.

Moreover, identifying teaching motivation as a mediator suggests the efficacy of motivational enhancement programs within educational institutions. Implementing professional development frameworks that foster intrinsic motivation among educators is imperative. Institutions can achieve this by acknowledging and valuing teachers’ contributions, offering autonomy in teaching methodologies, and creating pathways for professional growth and innovation. Collaborative opportunities that facilitate idea-sharing and platforms for peer recognition amplify teachers’ motivation and fulfillment within their roles, ultimately benefiting both educators and students.

These implications stress the urgency for systemic changes within educational institutions. Prioritizing teacher well-being and investing in initiatives that enhance their professional experiences are key. By acknowledging teachers as vital stakeholders and ensuring their emotional well-being, educational environments can be transformed into nurturing spaces that not only foster positive work cultures but also significantly influence the quality of education imparted. Ultimately, this focus on teachers positively impacts the academic success and holistic development of students.

### Limitations

While this study contributes significantly to the understanding of the relationships between teacher-student dynamics, perceived autonomy, teaching motivation, and emotional exhaustion, several limitations warrant acknowledgment. First, the cross-sectional nature of the study design restricts the establishment of definitive causal relationships between the variables investigated. Future longitudinal inquiries could offer valuable insights into the temporal order and causal pathways between teacher-student relationships, perceived autonomy, teaching motivation, and emotional exhaustion, providing a more robust foundation for understanding these dynamics.

Another limitation lies in the reliance on self-reported measures, which might introduce response biases and subjectivity into the data. Complementing quantitative assessments with qualitative investigations, such as interviews or observations, could offer richer insights into the multifaceted nature of teacher-student relationships, shedding light on nuanced aspects that quantitative measures might overlook. This methodological triangulation could enhance the depth and validity of findings, providing a more comprehensive understanding of the intricate dynamics between these variables.

Furthermore, the study’s focus on a specific educational context within a particular cultural setting might restrict the generalizability of the findings. Future research endeavors should aim to replicate and expand upon these investigations across diverse educational settings, cultural contexts, and geographical locations. Comparative studies across various educational systems and cultural backgrounds could elucidate how contextual factors influence the interrelationships between teacher-student dynamics, perceived autonomy, teaching motivation, and emotional well-being, allowing for a more nuanced understanding of these constructs in different environments.

### Suggestions for future research

Moving forward, future research could explore not only autonomy-supportive environments but also competence-supportive and relatedness-supportive environments within educational settings. Investigating how factors beyond autonomy, such as feelings of competence and connectedness, impact teacher well-being and motivation could enrich our understanding of the broader framework of Self-Determination Theory within the context of education. This expanded focus could involve examining how fostering feelings of competence in pedagogical skills and creating supportive social networks among educators influence emotional exhaustion and teaching motivation.

Additionally, exploring the impact of specific interventions or programs designed to promote competence and relatedness among educators within educational institutions could be a valuable area for future research (Ahmadi et al., 2023). These interventions might involve professional development strategies aimed at enhancing teachers’ perceived competence, collaborative practices to foster a sense of community and belonging among educators, or mentorship programs focused on promoting relatedness and support networks within teaching communities. Investigating the effects of such interventions on teacher well-being and motivation could offer practical insights for enhancing teacher support and fostering healthier educational environments.

Expanding research inquiries to encompass these dimensions beyond autonomy could offer a more comprehensive understanding of the multifaceted influences on teacher emotional well-being, motivation, and the quality of teacher-student relationships within educational settings.

## Data availability statement

The raw data supporting the conclusions of this article will be made available by the authors, without undue reservation. Requests to access these datasets should be directed to XW, Wangxy485nenu@163.com.

## Ethics statement

The studies involving humans were approved by College of Education Science, Harbin Normal University, Harbin, China. The studies were conducted in accordance with the local legislation and institutional requirements. The participants provided their written informed consent to participate in this study.

## Author contributions

XW: Conceptualization, Data curation, Formal analysis, Funding acquisition, Investigation, Methodology, Project administration, Resources, Software, Supervision, Validation, Visualization, Writing – original draft, Writing – review & editing. LY: Formal analysis, Investigation, Methodology, Project administration, Resources, Supervision, Visualization, Writing – original draft, Writing – review & editing. KC: Conceptualization, Data curation, Methodology, Resources, Software, Validation, Visualization, Writing – review & editing. YZ: Formal analysis, Investigation, Methodology, Resources, Software, Supervision, Visualization, Writing – review & editing.

## References

[ref1] Ahmadi A. Noetel M. Parker P. Ryan R. M. Ntoumanis N. Reeve J.. (2023). A classification system for teachers’ motivational behaviors recommended in self-determination theory interventions. J. Educ. Psychol. 115, 1158–1176. doi: 10.1037/edu0000783

[ref2] Amini M. A. Kruger C. G. (2022). The role of Iranian EFL teacher autonomy and reflectivity in teacher self-directed learning: a systematic literature review. Iran. J. Lang. Teach. Res. 10, 101–126. doi: 10.30466/ijltr.2022.121124

[ref3] Anderson J. C. Gerbing D. W. (1988). Structural equation modelling in practice: a review and recommended two-step approach. Psychol. Bull. 103, 411–423. doi: 10.1037/0033-2909.103.3.411

[ref4] Ansari A. Hofkens T. L. Pianta R. C. (2020). Teacher-student relationships across the first seven years of education and adolescent outcomes. J. Appl. Dev. Psychol. 71:101200. doi: 10.1016/j.appdev.2020.101200

[ref5] Baker J. A. Grant S. Morlock L. (2008). The teacher-student relationship as a developmental context for children with internalizing or externalizing behavior problems. Sch. Psychol. Q. 23, 3–15. doi: 10.1037/1045-3830.23.1.3

[ref6] Bardach L. Klassen R. M. Perry N. E. (2022). Teachers’ psychological characteristics: do they matter for teacher effectiveness, teachers’ well-being, retention, and interpersonal relations? An integrative review. Educ. Psychol. Rev. 34, 259–300. doi: 10.1007/s10648-021-09614-9

[ref7] Barfield V. Burlingame M. (1974). The pupil control ideology of teachers in selected schools. J. Exp. Educ. 42, 6–11. doi: 10.1080/00220973.1974.11011486

[ref8] Barni D. Danioni F. Benevene P. (2019). Teachers’ self-efficacy: the role of personal values and motivations for teaching. Front. Psychol. 10:1645. doi: 10.3389/fpsyg.2019.01645, PMID: 31354604 PMC6640028

[ref9] Bas G. (2022). Effect of student teachers’ teaching beliefs and attitudes towards teaching on motivation to teach: mediating role of self-efficacy. J. Educ. Teach. 48, 348–363. doi: 10.1080/02607476.2021.2006043

[ref10] Bing H. Sadjadi B. Afzali M. Fathi J. (2022). Self-efficacy and emotion regulation as predictors of teacher burnout among English as a foreign language teachers: a structural equation modeling approach. Front. Psychol. 13:900417. doi: 10.3389/fpsyg.2022.900417, PMID: 35664188 PMC9160989

[ref11] Carroll A. Forrest K. Sanders-O’Connor E. Flynn L. Bower J. M. Fynes-Clinton S.. (2022). Teacher stress and burnout in Australia: examining the role of intrapersonal and environmental factors. Soc. Psychol. Educ. 25, 441–469. doi: 10.1007/s11218-022-09686-7, PMID: 35233183 PMC8874312

[ref12] Chang M. L. (2009). An appraisal perspective of teacher burnout: examining the emotional work of teachers. Educ. Psychol. Rev. 21, 193–218. doi: 10.1007/s10648-009-9106-y

[ref13] Collie R. J. Bostwick K. C. Martin A. J. (2020). Perceived autonomy support, relatedness with students, and workplace outcomes: an investigation of differences by teacher gender. Educ. Psychol. 40, 253–272. doi: 10.1080/01443410.2019.1663791

[ref14] Collie R. J. Granziera H. Martin A. J. (2018). Teachers’ perceived autonomy support and adaptability: an investigation employing the job demands-resources model as relevant to workplace exhaustion, disengagement, and commitment. Teach. Teach. Educ. 74, 125–136. doi: 10.1016/j.tate.2018.04.015

[ref15] Corbin C. M. Alamos P. Lowenstein A. E. Downer J. T. Brown J. L. (2019). The role of teacher-student relationships in predicting teachers’ personal accomplishment and emotional exhaustion. J. Sch. Psychol. 77, 1–12. doi: 10.1016/j.jsp.2019.10.001, PMID: 31837719

[ref16] Cui L. (2022). The role of teacher–student relationships in predicting teachers’ occupational wellbeing, emotional exhaustion, and enthusiasm. Front. Psychol. 13:896813. doi: 10.3389/fpsyg.2022.896813 35664194 PMC9162152

[ref17] Deci E. L. Ryan R. M. (2000). The “what” and “why” of goal pursuits: human needs and the self-determination of behavior. Psychol. Inq. 11, 227–268. doi: 10.1207/S15327965PLI1104_01

[ref18] Deci E. L. Ryan R. M. Gagné M. Leone D. R. Usunov J. Kornazheva B. P. (2001). Need satisfaction, motivation, and well-being in the work organizations of a former eastern bloc country: a cross-cultural study of self-determination. Personal. Soc. Psychol. Bull. 27, 930–942. doi: 10.1177/0146167201278002

[ref19] Derakhshan A. Greenier V. Fathi J. (2023). Exploring the interplay between a loving pedagogy, creativity, and work engagement among EFL/ESL teachers: a multinational study. Curr. Psychol. 42, 22803–22822. doi: 10.1007/s12144-022-03371-w

[ref20] Dikilitaş K. Mumford S. E. (2019). Teacher autonomy development through reading teacher research: agency, motivation and identity. Innov. Lang. Learn. Teach. 13, 253–266. doi: 10.1080/17501229.2018.1442471

[ref21] Djonko-Moore C. M. (2022). Diversity education and early childhood teachers’ motivation to remain in teaching: an exploration. J. Early Childhood Teach. Educ. 43, 35–53. doi: 10.1080/10901027.2020.1806151

[ref22] Dong Y. Wang H. Luan F. Li Z. Cheng L. (2021). How children feel matters: teacher–student relationship as an indirect role between interpersonal trust and social adjustment. Front. Psychol. 11:581235. doi: 10.3389/fpsyg.2020.581235, PMID: 33536963 PMC7847853

[ref23] Dou D. Devos G. Valcke M. (2017). The relationships between school autonomy gap, principal leadership, teachers’ job satisfaction and organizational commitment. Educ. Manage. Admin. Leadership 45, 959–977. doi: 10.1177/1741143216653975

[ref24] Dymoke S. Harrison J. K. (2006). Professional development and the beginning teacher: issues of teacher autonomy and institutional conformity in the performance review process. J. Educ. Teach. 32, 71–92. doi: 10.1080/02607470500511009

[ref25] Eldor L. Shoshani A. (2016). Caring relationships in school staff: exploring the link between compassion and teacher work engagement. Teach. Teach. Educ. 59, 126–136. doi: 10.1016/j.tate.2016.06.001

[ref26] Evans D. Butterworth R. Law G. U. (2019). Understanding associations between perceptions of student behaviour, conflict representations in the teacher-student relationship and teachers’ emotional experiences. Teach. Teach. Educ. 82, 55–68. doi: 10.1016/j.tate.2019.03.008

[ref27] Fabris M. A. Lin S. Longobardi C. (2023). A cross-cultural comparison of teacher-student relationship quality in Chinese and Italian teachers and students. J. Sch. Psychol. 99:101227. doi: 10.1016/j.jsp.2023.101227, PMID: 37507185

[ref28] Falk D. Shephard D. Mendenhall M. (2022). “I always take their problem as mine”–understanding the relationship between teacher-student relationships and teacher well-being in crisis contexts. Int. J. Educ. Dev. 95:102670. doi: 10.1016/j.ijedudev.2022.102670

[ref29] Federici R. A. (2013). Principals’ self-efficacy: relations with job autonomy, job satisfaction, and contextual constraints. Eur. J. Psychol. Educ. 28, 73–86. doi: 10.1007/s10212-011-0102-5

[ref30] Fernet C. Guay F. Senécal C. Austin S. (2012). Predicting intraindividual changes in teacher burnout: the role of perceived school environment and motivational factors. Teach. Teach. Educ. 28, 514–525. doi: 10.1016/j.tate.2011.11.013

[ref31] Firestone W. A. (2014). Teacher evaluation policy and conflicting theories of motivation. Educ. Res. 43, 100–107. doi: 10.3102/0013189X14521864

[ref32] Fornell C. Larcker D. F. (1981). Evaluating structural equation models with un- observable variables and measurement error. J. Mark. Res. 18, 39–50. doi: 10.1177/002224378101800104

[ref33] Fradkin-Hayslip A. (2021). Teacher autonomy, motivation, and job satisfaction: perceptions of elementary school teachers according to self-determination theory. Ilkogretim Online 20. doi: 10.17051/ilkonline.2021.02.25

[ref34] Friedman I. A. (1999). Teacher-perceived work autonomy: the concept and its measurement. Educ. Psychol. Meas. 59, 58–76. doi: 10.1177/00131649921969749

[ref35] Frommelt M. Schiefele U. Lazarides R. (2021). Teacher enthusiasm, supportive instructional practices, and student motivation in mathematics classrooms. Interdiscip. Educ. Psychol. 2, 1–5. doi: 10.31532/InterdiscipEducPsychol.2.3.005

[ref36] Garcia-Rodriguez L. Redín C. I. Abaitua C. R. (2023). Teacher-student attachment relationship, variables associated, and measurement: a systematic review. Educ. Res. Rev. 38:100488. doi: 10.1016/j.edurev.2022.100488

[ref37] Gavrilyuk O. A. Loginova I. O. Buzovkina N. Y. (2013). Relations of perceived autonomy and burnout syndrome in university teachers. Int. J. Appl. Psychol. 3, 52–62. doi: 10.5923/j.ijap.20130303.04

[ref38] Gawlik M. A. (2005). Cutting loose: Autonomy and education in charter schools. University of California, Berkeley: Berkeley.

[ref39] Gawlik M. A. (2007). Beyond the charter schoolhouse door: teacher-perceived autonomy. Educ. Urban Soc. 39, 524–553. doi: 10.1177/0013124507304074

[ref40] Grayson J. L. Alvarez H. K. (2008). School climate factors relating to teacher burnout: a mediator model. Teach. Teach. Educ. 24, 1349–1363. doi: 10.1016/j.tate.2007.06.005

[ref41] Greenier V. Fathi J. Behzadpoor S. F. (2023). Teaching for creativity in an EFL context: the predictive roles of school climate, teaching enthusiasm, and metacognition. Think. Skills Creat. 50:101419. doi: 10.1016/j.tsc.2023.101419

[ref42] Haerens L. Matos L. Koc A. Benita M. Abos A. (2022). Examining school boards’ chaotic leadership style in relation to teachers’ job satisfaction and emotional exhaustion. Teach. Teach. Educ. 118:103821. doi: 10.1016/j.tate.2022.103821

[ref43] Hair J. Black W. Babin B. Anderson R. (2010). Multivariate data analysis 7th ed. Upper Saddle River, NJ, USA: Prentice-Hall

[ref44] Hakanen J. J. Bakker A. B. Schaufeli W. B. (2006). Burnout and work engagement among teachers. J. Sch. Psychol. 43, 495–513. doi: 10.1016/j.jsp.2005.11.001

[ref1002] Hamre B. K. Pianta R. C. (2001). Early teacher–child relationships and the trajectory of children’s school outcomes through eighth grade. Child Development. 72, 625–638. doi: 10.1111/1467-8624.00301 11333089

[ref45] Han Y. Kim Y. Hur W. M. (2021). The effects of perceived supervisor incivility on child-care workers’ job performance: the mediating role of emotional exhaustion and intrinsic motivation. Curr. Psychol. 40, 1979–1994. doi: 10.1007/s12144-019-0133-7

[ref46] Han J. Yin H. (2016). Teacher motivation: definition, research development and implications for teachers. Cogent Educ. 3:1217819. doi: 10.1080/2331186X.2016.1217819

[ref47] Hayes A. F. (2009). Beyond baron and Kenny: statistical mediation analysis in the new millennium. Commun. Monogr. 76, 408–420. doi: 10.1080/03637750903310360

[ref48] Heinz M. (2015). Why choose teaching? An international review of empirical studies exploring student teachers’ career motivations and levels of commitment to teaching. Educ. Res. Eval. 21, 258–297. doi: 10.1080/13803611.2015.1018278

[ref49] Helgøy I. Homme A. (2007). Towards a new professionalism in school? A comparative study of teacher autonomy in Norway and Sweden. Eur. Educ. Res. J. 6, 232–249. doi: 10.2304/eerj.2007.6.3.232

[ref50] Hoy W. K. (2001). The pupil control studies. A historical, theoretical and empirical analysis. J. Educ. Adm. 39, 424–441. doi: 10.1108/EUM0000000005812

[ref51] Hu L. T. Bentler P. M. (1999). Cutoff criteria for fit indexes in covariance structure analysis: conventional criteria versus new alternatives. Struct. Equ. Model. Multidiscip. J. 6, 1–55. doi: 10.1080/10705519909540118

[ref52] Hui Q. Yao C. Li M. You X. (2022). Upward social comparison sensitivity on teachers’ emotional exhaustion: a moderated moderation model of self-esteem and gender. J. Affect. Disord. 299, 568–574. doi: 10.1016/j.jad.2021.12.081, PMID: 34952113

[ref53] Hultell D. Gustavsson J. P. (2011). Factors affecting burnout and work engagement in teachers when entering employment. Work 40, 85–98. doi: 10.3233/WOR-2011-1209, PMID: 21849751

[ref54] Karpouza E. Emvalotis A. (2019). Exploring the teacher-student relationship in graduate education: a constructivist grounded theory. Teach. High. Educ. 24, 121–140. doi: 10.1080/13562517.2018.1468319

[ref55] Kiemer K. Gröschner A. Kunter M. Seidel T. (2018). Instructional and motivational classroom discourse and their relationship with teacher autonomy and competence support—findings from teacher professional development. Eur. J. Psychol. Educ. 33, 377–402. doi: 10.1007/s10212-016-0324-7

[ref56] Kleinkorres R. Stang-Rabrig J. McElvany N. (2023). The longitudinal development of students’ well-being in adolescence: the role of perceived teacher autonomy support. J. Res. Adolesc. 33, 496–513. doi: 10.1111/jora.12821, PMID: 36599803

[ref57] Kline R. B. (2023). Principles and practice of structural equation modeling. New York, NY: Guilford publications.

[ref58] Klusmann U. Aldrup K. Roloff-Bruchmann J. Carstensen B. Wartenberg G. Hansen J.. (2023). Teachers’ emotional exhaustion during the COVID-19 pandemic: levels, changes, and relations to pandemic-specific demands. Teach. Teach. Educ. 121:103908. doi: 10.1016/j.tate.2022.103908, PMID: 36247186 PMC9550665

[ref59] Klusmann U. Richter D. Lüdtke O. (2016). Teachers’ emotional exhaustion is negatively related to students’ achievement: evidence from a large-scale assessment study. J. Educ. Psychol. 108, 1193–1203. doi: 10.1037/edu0000125

[ref60] Koka A. Tilga H. Hein V. Kalajas-Tilga H. Raudsepp L. (2021). A multidimensional approach to perceived teachers’ autonomy support and its relationship with intrinsic motivation of students in physical education. Int. J. Sport Psychol. 52, 266–286. doi: 10.7352/IJSP.2021.52.266

[ref61] Kunter M. Frenzel A. Nagy G. Baumert J. Pekrun R. (2011). Teacher enthusiasm: dimensionality and context specificity. Contemp. Educ. Psychol. 36, 289–301. doi: 10.1016/j.cedpsych.2011.07.001

[ref62] Kunter M. Tsai Y. M. Klusmann U. Brunner M. Krauss S. Baumert J. (2008). Students’ and mathematics teachers’ perceptions of teacher enthusiasm and instruction. Learn. Instr. 18, 468–482. doi: 10.1016/j.learninstruc.2008.06.008

[ref63] Kyriacou C. (2001). Teacher stress: directions for future research. Educ. Rev. 53, 27–35. doi: 10.1080/00131910120033628

[ref64] La Guardia J. G. Ryan R. M. Couchman C. E. Deci E. L. (2000). Within-person variation in security of attachment: a self-determination theory perspective on attachment, need fulfillment, and well-being. J. Pers. Soc. Psychol. 79, 367–384. doi: 10.1037/0022-3514.79.3.367, PMID: 10981840

[ref65] Lamb T. (2000) in Learner autonomy, teacher autonomy: Future directions. eds. Sinclair B. McGrath I. (London: Longman).

[ref66] Lavy S. Bocker S. (2018). A path to teacher happiness? A sense of meaning affects teacher–student relationships, which affect job satisfaction. J. Happiness Stud. 19, 1485–1503. doi: 10.1007/s10902-017-9883-9

[ref67] Li F. Mohammaddokht F. Hosseini H. M. Fathi J. (2023). Reflective teaching and academic optimism as correlates of work engagement among university instructors. Heliyon 9:e13735. doi: 10.1016/j.heliyon.2023.e13735, PMID: 36865456 PMC9971167

[ref68] Liu L. Fathi J. Allahveysi S. P. Kamran K. (2023). A model of teachers’ growth mindset, teaching enjoyment, work engagement, and teacher grit among EFL teachers. Front. Psychol. 14:1137357. doi: 10.3389/fpsyg.2023.1137357, PMID: 36968701 PMC10030517

[ref69] Liu S. Keeley J. W. Sui Y. Sang L. (2021). Impact of distributed leadership on teacher job satisfaction in China: the mediating roles of teacher autonomy and teacher collaboration. Stud. Educ. Eval. 71:101099. doi: 10.1016/j.stueduc.2021.101099

[ref70] Longobardi C. Prino L. E. Marengo D. Settanni M. (2016). Student-teacher relationships as a protective factor for school adjustment during the transition from middle to high school. Front. Psychol. 7:1988. doi: 10.3389/fpsyg.2016.01988, PMID: 28066305 PMC5179523

[ref71] Longobardi C. Settanni M. Berchiatti M. Mastrokoukou S. Marengo D. (2023). Teachers’ sentiment about physical appearance of primary school students: associations with student–teacher relationship quality and student popularity among classroom peers. Soc. Psychol. Educ. 26, 383–403. doi: 10.1007/s11218-022-09749-9

[ref72] Longobardi C. Settanni M. Prino L. E. Fabris M. A. Marengo D. (2019). Students’ psychological adjustment in normative school transitions from kindergarten to high school: investigating the role of teacher-student relationship quality. Front. Psychol. 10:1238. doi: 10.3389/fpsyg.2019.01238, PMID: 31191415 PMC6548872

[ref73] Lyle A. M. Peurach D. J. (2022). Changing notions of teacher autonomy: the intersection of teacher autonomy and instructional improvement in the US. Res. Educ. 00345237211055843:003452372110558. doi: 10.1177/00345237211055843

[ref74] Martin A. J. Collie R. J. (2019). Teacher–student relationships and students’ engagement in high school: does the number of negative and positive relationships with teachers matter? J. Educ. Psychol. 111, 861–876. doi: 10.1037/edu0000317

[ref1003] Marsh H. W. Hau K. T. Wen Z. (2004). In search of golden rules: Comment on hypothesis-testing approaches to setting cutoff values for fit indexes and dangers in overgeneralizing Hu and Bentler’s (1999) findings. Structural Equation Modeling. 320–341.

[ref75] Maslach C. Jackson S. E. Leiter M. P. (1996). The Maslach burnout inventory manual. 3rd Edn. Palo Alto, CA: Consulting Psychologists Press.

[ref76] Maslach M. Leiter M. P. (1999). “Teacher burnout: a research agenda” in Understanding and preventing teacher burnout. eds. Huberman M. Vandenberghe R. (Cambridge, UK: Cambridge University Press), 295–303.

[ref77] Maslach C. Leiter M. P. (2016). Understanding the burnout experience: recent research and its implications for psychiatry. World Psychiatry 15, 103–111. doi: 10.1002/wps.20311, PMID: 27265691 PMC4911781

[ref78] Maslach C. Schaufeli W. B. Leiter M. P. (2001). Job burnout. Annu. Rev. Psychol. 52, 397–422. doi: 10.1146/annurev.psych.52.1.397 11148311

[ref79] Mérida-López S. Extremera N. (2017). Emotional intelligence and teacher burnout: a systematic review. Int. J. Educ. Res. 85, 121–130. doi: 10.1016/j.ijer.2017.07.006

[ref80] Murray C. Zvoch K. (2011). The inventory of teacher-student relationships: factor structure, reliability, and validity among African American youth in low-income urban schools. J. Early Adolesc. 31, 493–525. doi: 10.1177/0272431610366250

[ref81] Neter J. Kutner N. J. Nachtsheim C. V. Wasserman W. (1996). Applied linear statistical models 4th. Boston: Irwin.

[ref82] Nübling M. Stössel U. Hasselhorn H. M. Michaelis M. Hofmann F. (2006). Measuring psychological stress and strain at work: evaluation of the COPSOQ questionnaire in Germany. GMS Psycho Soc. Med. 3, 1–14. PMC2736502 19742072

[ref83] Olivier E. Lazariuk L. Archambault I. Morin A. J. (2023). Teacher emotional exhaustion: the synergistic roles of self-efficacy and student–teacher relationships. Soc. Psychol. Educ. 1-22. doi: 10.1007/s11218-023-09826-7

[ref84] Paré G. Tremblay M. (2007). The influence of high-involvement human resources practices, procedural justice, organizational commitment, and citizenship behaviors on information technology professionals’ turnover intentions. Group Org. Manag. 32, 326–357. doi: 10.1177/1059601106286875

[ref85] Parker G. (2015). Teachers’ autonomy. Res. Educ. 93, 19–33. doi: 10.7227/RIE.0008

[ref86] Patall E. A. Kennedy A. A. Yates N. Zambrano J. Lee D. Vite A. (2022). The relations between urban high school science students’ agentic mindset, agentic engagement, and perceived teacher autonomy support and control. Contemp. Educ. Psychol. 71:102097. doi: 10.1016/j.cedpsych.2022.102097

[ref87] Pearson L. C. Moomaw W. (2005). The relationship between teacher autonomy and stress, work satisfaction, empowerment, and professionalism. Educ. Res. Q. 29, 38–54.

[ref88] Pelletier L. G. Rocchi M. (2016). “Teachers’ motivation in the classroom” in Building autonomous learners. eds. Liu W. C. Wang J. C. K. Ryan R. M. (Singapore: Springer), 107–127. doi: 10.1007/978-981-287-630-0_6

[ref89] Pianta R. C. (1999). Enhancing relationships between children and teachers. Washington DC: American Psychological Association

[ref90] Pianta R. C. Hamre B. K. (2009). Conceptualization, measurement, and improvement of classroom processes: standardized observation can leverage capacity. Educ. Res. 38, 109–119. doi: 10.3102/0013189X09332374

[ref91] Podsakoff P. M. Organ D. W. (1986). Self-reports in organizational research: problems and prospects. J. Manag. 12, 531–544. doi: 10.1177/014920638601200408

[ref92] Pogere E. F. López-Sangil M. C. García-Señorán M. M. González A. (2019). Teachers’ job stressors and coping strategies: their structural relationships with emotional exhaustion and autonomy support. Teach. Teach. Educ. 85, 269–280. doi: 10.1016/j.tate.2019.07.001

[ref93] Poling D. V. Van Loan C. L. Garwood J. D. Zhang S. Riddle D. (2022). Enhancing teacher-student relationship quality: a narrative review of school-based interventions. Educ. Res. Rev. 37:100459. doi: 10.1016/j.edurev.2022.100459

[ref94] Poulou M. S. (2017). Social and emotional learning and teacher–student relationships: preschool teachers’ and students’ perceptions. Early Childhood Educ. J. 45, 427–435. doi: 10.1007/s10643-016-0800-3

[ref95] Robinson C. D. (2022). A framework for motivating teacher-student relationships. Educ. Psychol. Rev. 34, 2061–2094. doi: 10.1007/s10648-022-09706-0

[ref96] Roorda D. L. Jak S. Zee M. Oort F. J. Koomen H. M. (2017). Affective teacher–student relationships and students’ engagement and achievement: a meta-analytic update and test of the mediating role of engagement. Sch. Psychol. Rev. 46, 239–261. doi: 10.17105/SPR-2017-0035.V46-3

[ref97] Roorda D. L. Koomen H. M. Spilt J. L. Oort F. J. (2011). The influence of affective teacher–student relationships on students’ school engagement and achievement: a meta-analytic approach. Rev. Educ. Res. 81, 493–529. doi: 10.3102/0034654311421793

[ref98] Rumschlag K. E. (2017). Teacher burnout: a quantitative analysis of emotional exhaustion, personal accomplishment, and depersonalization. Int. Manage. Rev. 13:22.

[ref99] Ryan R. M. Deci E. (2014). “Self-determination theory” in Encyclopedia of quality of life and well-being research. ed. Michalos A. C. (Dordrecht: Springer), 5755–5760.

[ref100] Salokangas M. Wermke W. Harvey G. (2020). Teachers’ autonomy deconstructed: Irish and Finnish teachers’ perceptions of decision-making and control. Eur. Educ. Res. J. 19, 329–350. doi: 10.1177/1474904119868378

[ref101] Samfira E. M. Paloş R. (2021). Teachers’ personality, perfectionism, and self-efficacy as predictors for coping strategies based on personal resources. Front. Psychol. 12:751930. doi: 10.3389/fpsyg.2021.751930, PMID: 34795619 PMC8593193

[ref102] Samfira E. M. Sava F. A. (2021). Cognitive-behavioral correlates of pupil control ideology. PLoS One 16:e0246787. doi: 10.1371/journal.pone.0246787, PMID: 33566843 PMC7875416

[ref103] Sato M. Fernández Castillo F. Oyanedel J. C. (2022). Teacher motivation and burnout of English-as-a-foreign-language teachers: do demotivators really demotivate them? Front. Psychol. 13:891452. doi: 10.3389/fpsyg.2022.891452, PMID: 35572226 PMC9094067

[ref104] Skaalvik E. M. Skaalvik S. (2011). Teacher job satisfaction and motivation to leave the teaching profession: relations with school context, feeling of belonging, and emotional exhaustion. Teach. Teach. Educ. 27, 1029–1038. doi: 10.1016/j.tate.2011.04.001

[ref1001] Skaalvik E. M. Skaalvik S. (2013). School goal structure: Associations with students’ perceptions of their teachers as emotionally supportive, academic self-concept, intrinsic motivation, effort, and help seeking behavior. International Journal of Educational Research. 61, 5–14. doi: 10.1016/j.ijer.2013.03.007

[ref105] Skaalvik E. M. Skaalvik S. (2014). Teacher self-efficacy and perceived autonomy: relations with teacher engagement, job satisfaction, and emotional exhaustion. Psychol. Rep. 114, 68–77. doi: 10.2466/14.02.PR0.114k14w0, PMID: 24765710

[ref106] Skaalvik E. M. Skaalvik S. (2016). Teacher stress and teacher self-efficacy as predictors of engagement, emotional exhaustion, and motivation to leave the teaching profession. Creat. Educ. 7, 1785–1799. doi: 10.4236/ce.2016.713182

[ref107] Skaalvik E. M. Skaalvik S. (2017). Motivated for teaching? Associations with school goal structure, teacher self-efficacy, job satisfaction and emotional exhaustion. Teach. Teach. Educ. 67, 152–160. doi: 10.1016/j.tate.2017.06.006

[ref108] Skaalvik E. M. Skaalvik S. (2020). Teacher burnout: relations between dimensions of burnout, perceived school context, job satisfaction and motivation for teaching. A longitudinal study. Teach. Teach 26, 602–616. doi: 10.1080/13540602.2021.1913404

[ref109] Skaalvik E. M. Skaalvik S. (2021). Collective teacher culture: exploring an elusive construct and its relations with teacher autonomy, belonging, and job satisfaction. Soc. Psychol. Educ. 24, 1389–1406. doi: 10.1007/s11218-021-09673-4

[ref110] Spilt J. L. Koomen H. M. Thijs J. T. (2011). Teacher wellbeing: the importance of teacher–student relationships. Educ. Psychol. Rev. 23, 457–477. doi: 10.1007/s10648-011-9170-y

[ref111] Stefanou C. R. Perencevich K. C. DiCintio M. Turner J. C. (2004). Supporting autonomy in the classroom: ways teachers encourage student decision making and ownership. Educ. Psychol. 39, 97–110. doi: 10.1207/s15326985ep3902_2

[ref112] Tabachnick B. G. Fidell L. S. Ullman J. B. (2013). Using multivariate statistics 6, 497–516. Boston, MA: Pearson.

[ref113] Taxer J. L. Becker-Kurz B. Frenzel A. C. (2019). Do quality teacher–student relationships protect teachers from emotional exhaustion? The mediating role of enjoyment and anger. Soc. Psychol. Educ. 22, 209–226. doi: 10.1007/s11218-018-9468-4

[ref114] Tian J. Zhang W. Mao Y. Gurr D. (2022). The impact of transformational leadership on teachers’ job burnout: the mediating role of social-emotional competence and student-teacher relationship. J. Educ. Adm. 60, 369–385. doi: 10.1108/JEA-04-2021-0075

[ref115] Tilga H. Hein V. Koka A. (2017). Measuring the perception of the teachers’ autonomy supportive behavior in physical education: development and initial validation of a multidimensional instrument. Meas. Phys. Educ. Exerc. Sci. 21, 244–255. doi: 10.1080/1091367X.2017.1354296

[ref116] Tilga H. Kalajas-Tilga H. Hein V. Raudsepp L. Koka A. (2021). Effects of a web-based autonomy-supportive intervention on physical education teacher outcomes. Educ. Sci. 11:316. doi: 10.3390/educsci11070316

[ref117] Van den Berghe L. Cardon G. Aelterman N. Tallir I. B. Vansteenkiste M. Haerens L. (2013). Emotional exhaustion and motivation in physical education teachers: a variable-centered and person-centered approach. J. Teach. Phys. Educ. 32, 305–320. doi: 10.1123/jtpe.32.3.305

[ref118] Vangrieken K. Grosemans I. Dochy F. Kyndt E. (2017). Teacher autonomy and collaboration: a paradox? Conceptualising and measuring teachers’ autonomy and collaborative attitude. Teach. Teach. Educ. 67, 302–315. doi: 10.1016/j.tate.2017.06.021

[ref119] Vansteenkiste M. Simons J. Lens W. Sheldon K. M. Deci E. L. (2004). Motivating learning, performance, and persistence: the synergistic effects of intrinsic goal contents and autonomy-supportive contexts. J. Pers. Soc. Psychol. 87, 246–260. doi: 10.1037/0022-3514.87.2.246, PMID: 15301630

[ref120] Vermote B. Aelterman N. Beyers W. Aper L. Buysschaert F. Vansteenkiste M. (2020). The role of teachers’ motivation and mindsets in predicting a (de) motivating teaching style in higher education: a circumplex approach. Motiv. Emot. 44, 270–294. doi: 10.1007/s11031-020-09827-5

[ref121] Wang Q. Zhang H. (2014). Promoting teacher autonomy through university–school collaborative action research. Lang. Teach. Res. 18, 222–241. doi: 10.1177/1362168813505942

[ref122] Watt H. M. Richardson P. W. (2008). Motivations, perceptions, and aspirations concerning teaching as a career for different types of beginning teachers. Learn. Instr. 18, 408–428. doi: 10.1016/j.learninstruc.2008.06.002

[ref123] Wentzel K. R. (2009). “Students’ relationships with teachers as motivational contexts” in Handbook of motivation at school. eds. Wentzel K. R. Wigfield A. Malwah, NJ: Erlbaum, 301–322.

[ref124] Wermke W. Höstfält G. (2014). Contextualizing teacher autonomy in time and space: a model for comparing various forms of governing the teaching profession. J. Curric. Stud. 46, 58–80. doi: 10.1080/00220272.2013.812681

[ref126] Wong M. Y. (2016). Teacher–student power relations as a reflection of multileveled intertwined interactions. Br. J. Sociol. Educ. 37, 248–267. doi: 10.1080/01425692.2014.916600

[ref127] Xia J. Wang M. Zhang S. (2022). School culture and teacher job satisfaction in early childhood education in China: the mediating role of teaching autonomy. Asia Pac. Educ. Rev. 24, 101–111. doi: 10.1007/s12564-021-09734-5

[ref128] Yin H. Huang S. Chen G. (2019). The relationships between teachers’ emotional labor and their burnout and satisfaction: a meta-analytic review. Educ. Res. Rev. 28:100283. doi: 10.1016/j.edurev.2019.100283

[ref129] Zhang L. J. Fathi J. Mohammaddokht F. (2023). Predicting teaching enjoyment from teachers’ perceived school climate, self-efficacy, and psychological wellbeing at work: EFL teachers. Percept. Mot. Skills 130, 2269–2299. doi: 10.1177/00315125231182269, PMID: 37395156 PMC10552353

